# Adenosine A_2B_ Receptor: From Cell Biology to Human Diseases

**DOI:** 10.3389/fchem.2016.00037

**Published:** 2016-08-24

**Authors:** Ying Sun, Pingbo Huang

**Affiliations:** ^1^Department of Biology, South University of Science and Technology of ChinaShenzhen, China; ^2^Shenzhen Key Laboratory of Cell Microenvironment, South University of Science and Technology of ChinaShenzhen, China; ^3^Division of Life Science, Hong Kong University of Science and TechnologyHong Kong, China; ^4^Division of Biomedical Engineering, Hong Kong University of Science and TechnologyHong Kong, China; ^5^State Key Laboratory of Molecular Neuroscience, Hong Kong University of Science and TechnologyHong Kong, China

**Keywords:** A_2B_ adenosine receptor, binding proteins, cancer, renal disease, diabetes

## Abstract

Extracellular adenosine is a ubiquitous signaling molecule that modulates a wide array of biological processes. Recently, significant advances have been made in our understanding of A_2B_ adenosine receptor (A_2B_AR). In this review, we first summarize some of the general characteristics of A_2B_AR, and then we describe the multiple binding partners of the receptor, such as newly identified α-actinin-1 and p105, and discuss how these associated proteins could modulate A_2B_AR's functions, including certain seemingly paradoxical functions of the receptor. Growing evidence indicates a critical role of A_2B_AR in cancer, renal disease, and diabetes, in addition to its importance in the regulation of vascular diseases, and lung disease. Here, we also discuss the role of A_2B_AR in cancer, renal disease, and diabetes and the potential of the receptor as a target for treating these three diseases.

## Introduction

Extracellular adenosine is a ubiquitous signaling molecule that modulates a wide array of biological processes. Most of the extracellular adenosine is derived from the release and metabolism of adenine nucleotides such as ATP following diverse stimuli, including mechanical stress, osmotic challenge, inflammation, and tissue injury (Dunwiddie et al., 1997; Fredholm et al., 2001a; Picher et al., 2003, 2004; Eckle et al., 2007; Grenz et al., 2007; Ohta and Sitkovsky, 2014; Ross et al., 2014; Fuentes and Palomo, 2015; Kowal et al., 2015; Borea et al., 2016; Covarrubias et al., 2016; Hamidzadeh and Mosser, 2016). Conversely, extracellular adenosine is eliminated mainly through two mechanisms: one, transport of adenosine back into the cell by nucleoside transporters; and two, deamination of adenosine to inosine by adenosine deaminase (ADA; Blackburn and Kellems, 1996) or phosphorylation of adenosine to AMP by adenosine kinase (Lloyd and Fredholm, 1995; Spychala et al., 1996). The combined actions of these adenosine generation and elimination mechanisms regulate extracellular adenosine levels, which range from 10 to 200 nM under homeostatic conditions but can be elevated to 10–100 μM in hypoxic or stressed environments (Fredholm, 2007).

The biological functions of extracellular adenosine are mediated by four subtypes of adenosine receptors (ARs), A_1_, A_2A_, A_2B_, and A_3_, each of which presents a unique pharmacological profile, tissue distribution, and effector coupling (Fredholm et al., 2001b). Among human ARs, A_1_AR, and A_3_AR share 49% sequence similarity and A_2A_AR and A_2B_AR share 59% similarity (Jacobson and Gao, 2006; Goblyos and Ijzerman, 2009).

Perhaps because A_2B_AR binds to adenosine with low affinity (EC_50_ = 24 μM; Beukers et al., 2000; Fredholm et al., 2001b, 2011a), A_2B_AR is frequently considered to represent a low-affinity version of A_2A_AR and to be of comparatively lesser physiological relevance. However, recent advances in pharmacological and molecular tools have allowed researchers to determine that A_2B_AR can be coupled to distinct intracellular signaling pathways and play physiological roles that differ from those of A_2A_AR (Yang et al., 2006, 2010a; Grenz et al., 2012a; Johnston-Cox et al., 2012; Koupenova et al., 2012; Eckle et al., 2013; Morello and Miele, 2014; Patel et al., 2014; Tak et al., 2014; Eisenstein et al., 2015; Tang et al., 2015; Vecchio et al., 2016). In this review, we discuss our current understanding of the cellular functions of A_2B_AR and their implications for the pathogenesis of several human diseases.

## Molecular function and cellular localization of A_2B_AR

A_2B_AR was first identified and cloned in 1992 by Rivkees and Reppert and by Pierce et al. from the rat hypothalamus (Rivkees and Reppert, 1992) and human hippocampus (Pierce et al., 1992). The proposed structure of A_2B_AR is the typical G-protein-coupled receptor (GPCR) structure, and the predicted molecular mass of A_2B_AR is 36–37 kDa (Feoktistov and Biaggioni, 1997).

The major signaling pathway of A_2B_AR is suggested to be the pathway involving adenylyl cyclase (AC) that leads to an increase in intracellular cAMP levels and results in the subsequent activation of PKA and other cAMP effectors such as Epac (Peakman and Hill, 1994; Murakami et al., 2000; Sitaraman et al., 2001; Lynge et al., 2003; Fang and Olah, 2007; Darashchonak et al., 2014; He et al., 2014). However, the A_2B_AR-Gq-PLC pathway also mediates several crucial functions of A_2B_AR (Gao et al., 1999; Linden et al., 1999; Panjehpour et al., 2005), and A_2B_AR further couples to the MAPK and arachidonic acid signaling pathways and regulates membrane ion channels probably through G-protein βγ subunits (Feoktistov et al., 1999; Jimenez et al., 1999; Schulte and Fredholm, 2003a,b; Donoso et al., 2005).

The recent development of A_2B_AR-knockout/lacZ-knockin mice has enabled the determination of A_2B_AR distribution *in vivo* (Yang et al., 2006); A_2B_AR is widely expressed in numerous tissues and organs, including the vasculature, aortic vascular smooth muscle, cecum, large intestine, brain, and urinary bladder (Yaar et al., 2005; Wang and Huxley, 2006; Yang et al., 2006). Furthermore, a high level of A_2B_AR expression has been detected in diverse types of cells, including various immune cells such as mast cells (Hua et al., 2007; Ryzhov et al., 2008b), neutrophils (Eckle et al., 2008a), dendritic cells (Pacheco et al., 2005; Ben Addi et al., 2008; Novitskiy et al., 2008), macrophages (Yang et al., 2006), and lymphocytes (Mirabet et al., 1999; Eckle et al., 2008a), as well as other cell types such as type II alveolar epithelial cells (Cagnina et al., 2009), endothelial cells (Yang et al., 2006), chromaffin cells (Casado et al., 1992), astrocytes (Peakman and Hill, 1994; Jimenez et al., 1999), neurons (Corset et al., 2000; Christofi et al., 2001; Stein et al., 2001), and taste cells (Nishida et al., 2014). Moreover, A_2B_AR expression is influenced by diverse environmental cues such as inflammation, cell stress, injury, and hypoxia (Xaus et al., 1999; Fredholm et al., 2001a; Kolachala et al., 2005; Kong et al., 2006; Hart et al., 2009; Hasko et al., 2009). For example, previous studies have shown that interferon-γ, a proinflammatory cytokine, increases the A_2B_AR transcriptional level in mouse macrophage cells (Xaus et al., 1999); TNF-α upregulates A_2B_AR mRNA and protein levels in human colonic epithelial cells (Kolachala et al., 2005); and other mediators such as LPS (Nemeth et al., 2003), IL-1β (Nguyen et al., 2003), free radicals (St Hilaire et al., 2008), and endogenous adenosine (Sitaraman et al., 2002) also enhance A_2B_AR expression.

## A_2B_AR binding partners and their cellular functions

**Figure 1:**
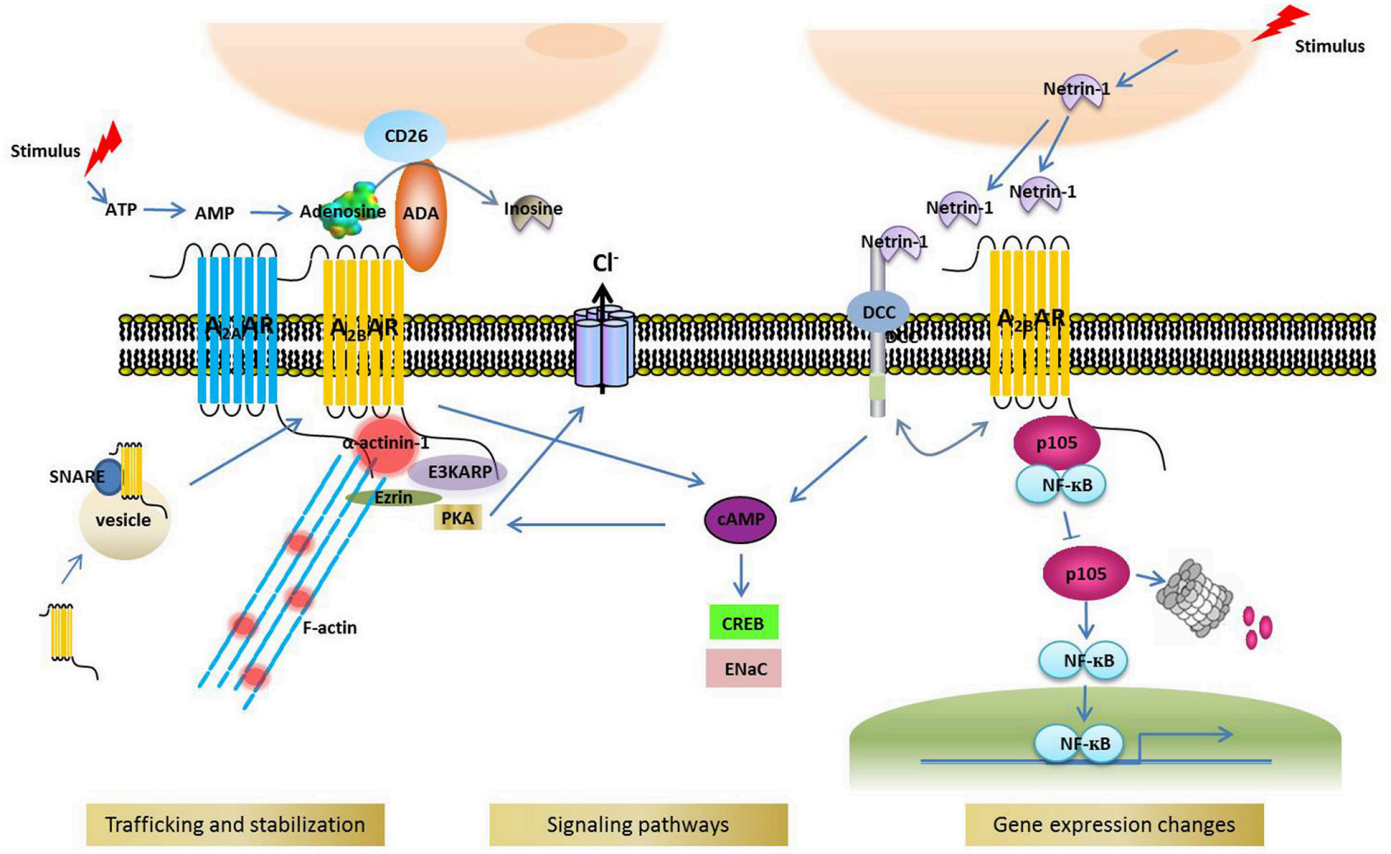
**A_2B_AR binding partners and their cellular functions**.

Identifying the binding partners of A_2B_AR is crucial for understanding the receptor's function and regulation. As in other GPCRs, the intracellular portions of A_2B_AR serve as signal integrators by providing binding sites for effectors or regulatory proteins, although other parts of A_2B_AR might also be involved in protein interaction. Besides trimeric G proteins and β-arrestin (Feoktistov and Biaggioni, 1997; Mundell et al., 2000; Klinger et al., 2002), the two universal binding partners of GPCRs, numerous other proteins interact with A_2B_AR. Here, we list these A_2B_AR binding partners in the order of interaction discovery, and discuss how these proteins modulate or mediate A_2B_AR functions (Figure 1).

## ADA

ADA is an enzyme that catalyzes the hydrolytic deamination of adenosine to inosine. Apart from being present in the cytosol and the nucleus, ADA is anchored to the cell surface by other membrane proteins, including CD26 (Pacheco et al., 2005) and A_1_AR (Saura et al., 1998) in various cell/tissue types such as cultured cortical neurons (Ruiz et al., 2000), DDT1MF-2 cells (Ciruela et al., 1996), and pig brain cortical membrane (Saura et al., 1996). In addition to A_1_AR and CD26, A_2B_AR was reported to mediate ADA docking—in CHO and Jurkat cells—onto the extracellular surface (Herrera et al., 2001); counterintuitively, the binding of ADA, even when ADA lacked enzymatic activity, increased the binding affinity of NECA (a nonselective A_2_AR agonist) for A_2B_AR and the subsequent production of cAMP. The interaction between ADA and A_2B_AR was also confirmed in dendritic cells (Pacheco et al., 2005) and gastric mucosa parietal cells (Arin et al., 2015). In dendritic cells, the ADA-A_2B_AR complex triggers a cell adhesion-costimulatory signal that promotes an immune response, and this is also independent of ADA enzymatic activity (Pacheco et al., 2005). Thus, the ADA-A_2B_AR complex appears to perform multiple functions, including modulating agonist binding, promoting cell adhesion/costimulation, and degrading extracellular adenosine.

## Deleted in colorectal carcinoma (DCC) and netrin-1

DCC has been proposed to function as a netrin-1 receptor and thus mediate netrin-1-induced axon outgrowth. Corset and collaborators identified A_2B_AR as one of the proteins that directly binds to DCC and functions as a netrin-1 coreceptor, because netrin-1 activated A_2B_AR and induced cAMP production, and further suggested that A_2B_AR is the central mediator of netrin signaling in the regulation of the outgrowth of dorsal spinal cord axons (Corset et al., 2000). However, a subsequent study argued against this view (Stein et al., 2001): the DCC ectodomain was found to interact directly with netrin-1 and mediate netrin signaling to regulate axon growth, and the results of pharmacological analyses suggested that A_2B_AR function was not required for netrin-1-induced axon growth and guidance. Thus, DCC was proposed to mediate netrin signaling in axon growth and guidance independently of A_2B_AR activation (Stein et al., 2001). Intriguingly, more recent studies have reported that netrin-1 attenuates neutrophil transmigration and hypoxia-induced inflammation (Rosenberger et al., 2009), alveolar fluid clearance (He et al., 2014), and diabetic nephropathy (Tak et al., 2013) and induces cancer-cell invasion (Rodrigues et al., 2007) in an A_2B_AR-dependent manner. These results appear to support the general notion that A_2B_AR mediates the function of netrin-1 at least in certain tissues. Further investigation is required to clarify the discrepancy between the aforementioned studies.

## E3KARP-ezrin-PKA and snare

Sitaraman and colleagues demonstrated that the majority of A_2B_AR localizes intracellularly in quiescent cells and is recruited to the plasma membrane upon agonist stimulation (Sitaraman et al., 2002). The SNARE protein SNAP-23 directly interacts with human A_2B_AR and participates in A_2B_AR recruitment to the plasma membrane (Wang et al., 2004), and following SNARE-dependent translocation to the plasma membrane, human A_2B_AR directly associates with E3KARP (NHERF2) and ezrin and forms a multiprotein complex (Sitaraman et al., 2002). Ezrin is a PKA-anchoring protein, or AKAP, that associates with the actin cytoskeleton (Sun et al., 2000), and this multiprotein complex not only anchors A_2B_AR to the plasma membrane, but also stabilizes A_2B_AR expression in the plasma membrane. Furthermore, compartmentalized PKA is effectively activated by A_2B_AR-induced cAMP production, and the PKA thus activated stimulates CFTR-mediated chloride secretion; this model is consistent with the functional evidence obtained in an early study (Huang et al., 2001).

Interestingly, at its C-terminal end, human A_2B_AR contains a type 2 PDZ-binding motif (XΦXΦ), GVGL, but not a type 1 PDZ-binding motif (XS/TXV/L). Sitaraman et al. speculated that a PDZ-binding-motif-like sequence in the 3rd intracellular loop in A_2B_AR might mediate the interaction with E3KARP, a PDZ-domain-containing protein (Sitaraman et al., 2002). However, recent studies indicate that the GVGL sequence of A_2B_AR participates in the trafficking and surface expression of A_2B_AR (Watson et al., 2011, 2016), possibly by binding to a PDZ-domain-containing protein. Further investigation is required to determine whether GVGL binds to E3KARP or another PDZ-domain-containing protein.

## A_2A_AR

The function and trafficking of several GPCRs are affected by the heterooligomerization of these receptors. Moriyama and Sitkovsky reported that A_2A_AR coexpression with A_2B_AR improves the cell-surface expression of A_2B_AR, which is normally poor because A_2B_AR lacks a dominant forward-transport signal for export from the ER to the cell surface (Moriyama and Sitkovsky, 2010). The study further suggested that the functional interaction between A_2A_AR and A_2B_AR might be a consequence of their physical association (Moriyama and Sitkovsky, 2010), but how these two receptors interact was not explored. Because both A_2A_AR and A_2B_AR were shown to interact with actinins in one previous study (in which the specific actinin isoform was not identified; Burgueno et al., 2003) or with α-actinin-1 in another study (Sun et al., 2016), the α-actinin-1 homodimer or a heterodimer of α-actinin-1 with another actinin isoform might mediate the dimerization of A_2A_AR and A_2B_AR and thus promote the surface expression of A_2B_AR. This mechanism is clearly not mutually exclusive with the mechanism by which α-actinin-1 mediates A_2B_AR interaction with actin filaments and thereby modulates the trafficking and surface expression of A_2B_AR (Sun et al., 2016).

## Transcription factor NFκB1/p105

NFκB1/p105 is a member of the NFκB family of proteins that perform regulatory functions in diverse biological processes such as inflammation and cell survival and differentiation, as well as in various diseases, including cancer (Barkett and Gilmore, 1999; Hatada et al., 2000; Perkins and Gilmore, 2006). Sun et al. reported that the C-terminal tail of A_2B_AR binds to NFκB1/p105 independently of ligand activation (Sun et al., 2012). Intriguingly, A_2B_AR binding to specific sites on p105 prevents the polyubiquitination and degradation of p105 protein and thereby inhibits NFκB activation and reduces inflammation (Sun et al., 2012). In previous studies, both pro- and anti-inflammatory activities have been associated with A_2B_AR (Blackburn et al., 2009), and the work by Sun et al. potentially sheds light on this paradox: although A_2B_AR activation by adenosine produces proinflammatory effects, A_2B_AR can also induce adenosine-independent downregulation of the proinflammatory response by associating with p105. Such receptor bifunctionality displayed by A_2B_AR—mediation of diametrically opposite effects in the presence and absence of ligand—is reminiscent of dependence receptors (Thibert and Fombonne, 2010). GPCRs other than A_2B_AR have previously been shown to signal through G-protein-independent pathways, including pathways involving transcription factors (Nehring et al., 2000; White et al., 2000). The study of Sun et al. further suggests that the C-terminus of A_2B_AR potentially provides a target for developing peptidemimetic drugs that block NFκB signaling, which could be used for treating NFκB-related diseases such as inflammation and cancer (Sun et al., 2012).

## α-actinin-1

Actinins, or α-actinins, represent a family of ubiquitously expressed actin-filament-crosslinking proteins. In addition to performing their critical function of actin-filament crosslinking, actinins link membrane receptors, and cell adhesion proteins to actin filaments and thereby modulate the function and trafficking of these membrane proteins (Oikonomou et al., 2011; Foley and Young, 2014). A recent study by Sun and colleagues suggested that α-actinin-1 binds to the A_2B_AR C-terminus and stabilizes the receptor's global and cell-surface expression (Sun et al., 2016), which revealed a previously unidentified molecular mechanism for controlling the cellular levels of A_2B_AR. Because the actinin-1 isoform investigated in the study was the Ca^2+^-sensitive exon19a splice variant, an intriguing question is whether actinin-1-dependent regulation of A_2B_AR is also Ca^2+^ sensitive under physiological conditions.

In contrast to α-actinin-1, actinin-4, another highly homologous non-muscle actinin isoform, did not interact with A_2B_AR (Sun et al., 2016). Interestingly, actinin-4 has been suggested to interact with the NFκB subunits p65 and p50 and function as a coactivator of the transcription factor NFκB (Zhao et al., 2015). Thus, future studies could investigate whether actinin-1 also associates with NFκB proteins, including p105, and how this association affects the interaction between p105 and A_2B_AR.

## A_2B_AR in human diseases

**Figure 2:**
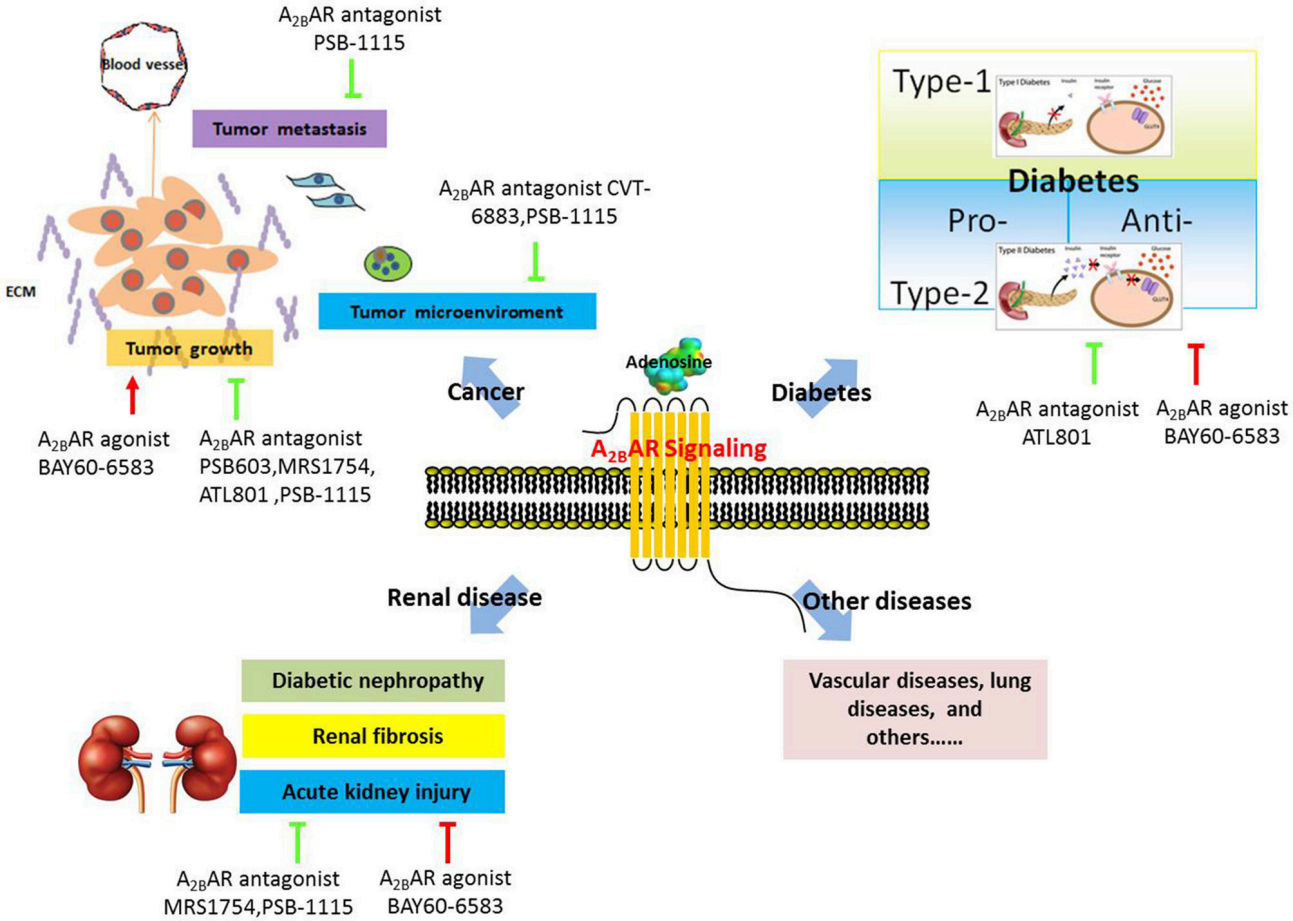
**Schematic presentation of the role of A_2B_AR in various human diseases**.

Numerous studies have demonstrated a critical role of A_2B_AR in the regulation of vascular diseases (Martin, 1992; Dubey et al., 1996; Yang et al., 2008, 2010a), chronic lung disease (Sun et al., 2006; Wilson et al., 2009; Zhou et al., 2009; Zaynagetdinov et al., 2010), and acute lung injury (Eckle et al., 2008a,b; Schingnitz et al., 2010), and several excellent reviews have summarized these studies (Spicuzza et al., 2006; Hasko et al., 2009; Aherne et al., 2011; Headrick et al., 2013). Therefore, in this review, we discuss only the potential functions of A_2B_AR in three other common human diseases, cancer, renal disease, and diabetes (Figure 2).

## A_2B_AR in cancer

Growing evidence indicates that A_2B_AR potentially plays a pathophysiological role in human cancer and might serve as a target for novel therapies or cotherapies for cancer. The possible functions of A_2B_AR in tumor progression and metastasis are discussed here.

First, A_2B_AR is highly expressed in various types of tumor cells or tissues and promotes tumor-cell proliferation. For instance, A_2B_AR was found to be overexpressed in colorectal carcinoma cells and tissues, and inhibition of A_2B_AR blocked the proliferation of colon cancer cells (Ma et al., 2010). In prostate cancer, A_2B_AR increased cancer-cell proliferation in both ligand-dependent, and ligand-independent manners (Wei et al., 2013; Vecchio et al., 2016). In human oral cancer, A_2B_AR was shown to be upregulated in oral squamous carcinoma cells, and A_2B_AR knockdown reduced the proliferation of oral cancer cells through HIF-1α activation (Kasama et al., 2015). Moreover, A_2B_AR was reported to foster bladder and breast tumor growth in syngeneic mice (Cekic et al., 2012).

Second, A_2B_AR modulates tumor-cell metastasis. A_2B_AR was implicated in promoting breast cancer cell migration *in vitro* and lung metastasis *in vivo* (Stagg et al., 2010; Desmet et al., 2013), although the underlying molecular mechanism was not fully elucidated. However, the results of a subsequent study suggested a possible explanation: A_2B_AR activation suppressed the prenylation of the small GTPase Rap1B and diminished Rap1B-mediated cell adhesion, which promoted cell migration (Ntantie et al., 2013).

Third, A_2B_AR might regulate the tumor microenvironment, including the surrounding blood vessels, immune cells, fibroblasts, and the extracellular matrix. Ryzhov and colleagues provided the first genetic evidence indicating that A_2B_AR regulates vascular endothelial growth factor (VEGF) production from tumor-infiltrating host immune cells and thereby promotes tumor growth (Ryzhov et al., 2008a). Concomitantly, other groups suggested that A_2B_AR alters angiogenesis by regulating the production of a wide array of pro- or anti-angiogenic factors such as basic fibroblast growth factor (bFGF), angiopoietin2, and a subset of cytokines (Feoktistov et al., 2002, 2003; Merighi et al., 2009). In addition to affecting angiogenesis, A_2B_AR regulates dendritic-cell differentiation and function (Novitskiy et al., 2008; Yang et al., 2010b) and alternative macrophage activation (Csoka et al., 2012) and thus contributes to cancer progression.

Thus, A_2B_AR exerts various effects on tumor progression and metastasis. Notably, most of the aforementioned evidence was collected using *in vitro* systems, and it is critical to further confirm the role of A_2B_AR in cancer by using *in vivo* models before A_2B_AR is used as a potential cancer therapeutic target.

## A_2B_AR in renal disease

Renal diseases are estimated to affect millions of people worldwide, whose numbers are growing at a rate of approximately 5–8% annually (Hamer and El Nahas, 2006). Several studies have indicated a critical role of A_2B_AR in mediating the progression of diabetic nephropathy. Patel et al. and Valladares et al. observed that inhibition of A_2B_AR activation suppressed VEGF production in glomeruli and further attenuated renal dysfunction in diabetic nephropathy; these data suggested a protective role of A_2B_AR antagonists in VEGF-induced diabetic nephropathy (Valladares et al., 2008; Patel and Thaker, 2014). However, this view was challenged by Tak et al., who reported elevated VEGF levels in diabetic A_2B_AR-knockout mice (Tak et al., 2014); concordantly, diabetic nephropathy was highly severe in mice with global or vascular endothelial tissue-specific A_2B_AR deletion, but not in mice with tubular-epithelial A_2B_AR deletion. Therefore, Tak et al. suggested that vascular A_2B_AR signaling is the key mediator of kidney protection during diabetic nephropathy (Tak et al., 2014). The methods used and the specific tissues studied by the aforementioned groups were distinct, which might explain their conflicting observations on the role of A_2B_AR during diabetic nephropathy. Moreover, the different time windows in which A_2B_AR inhibition was induced pharmacologically and genetically might also contribute to the discrepancy in the results (Eisenstein et al., 2015).

In addition to playing a role in diabetic nephropathy, A_2B_AR has been suggested, based on studies on several mouse models, to protect against renal fibrosis. In ADA-deficient mice, a high level of adenosine in kidney tissues resulted in proteinuria and renal fibrosis, and treatment with A_2B_AR antagonists attenuated renal dysfunction and fibrosis (Dai et al., 2011). Moreover, genetic deletion of A_2B_AR protected against renal fibrosis in both mice infused with angiotensin II and mice subjected to unilateral ureteral obstruction (Dai et al., 2011). Furthermore, renal biopsy samples from patients with chronic kidney disease (CKD) showed higher levels of A_2B_AR expression than did samples from patients without CKD (Zhang et al., 2013). All of these data suggest that A_2B_AR could serve as a potential therapeutic target in the treatment of CKD.

Acute kidney injury, a devastating kidney disease, is often caused by renal ischemia. Rigorous studies from different laboratories have suggested a pivotal role of A_2B_AR in acute kidney injury. For example, Grenz et al. used genetic and pharmacological approaches to reveal a role of A_2B_AR in protecting against renal injury resulting from ischemia, although the underlying molecular mechanism was not fully clarified (Grenz et al., 2008). Subsequently, the same group proposed two possible explanations for how A_2B_AR might provide renal protection: one, A_2B_AR reduces neutrophil-dependent TNF-α production and suppresses inflammation (Grenz et al., 2012b); and two, A_2B_AR promotes optimal postischemic blood flow within the kidney and thereby ensures the maximal return of blood flow, tissue oxygenation, and removal of waste products from the ischemic kidney through the A_2B_AR-ENT1 (equilibrative nucleoside transporter) pathway (Grenz et al., 2012a).

## A_2B_AR in diabetes

Diabetes mellitus (DM) is the most common endocrine disorder; in 2014, 9% of all adults aged 18+ years were estimated to have diabetes (WHO, 2014), and by 2025, 300 million people worldwide will have the disease (Mane et al., 2012). Adenosine has long been recognized to affect insulin secretion and glucose homeostasis by activating the four AR subtypes (Dong et al., 2001; Nemeth et al., 2007; Fredholm et al., 2011b; Koupenova and Ravid, 2013; Andersson, 2014; Antonioli et al., 2015). Recently, A_2B_AR in particular has been suggested to function as a critical regulator in DM (Rusing et al., 2006; Johnston-Cox et al., 2012, 2014; Eisenstein et al., 2015; Merighi et al., 2015; Wen et al., 2015).

In a type I DM model, the nonselective receptor agonist NECA blocked diabetes development, and this appeared to be mediated by A_2B_AR-dependent suppression of proinflammatory cytokine production (Nemeth et al., 2007). These data suggest that A_2B_AR represents a potential target for the treatment of type I diabetes.

Conversely, some of the evidence obtained using a type II DM model indicated that A_2B_AR plays a pro-diabetic role. Figler et al. suggested that A_2B_AR activation increases insulin resistance by elevating the production of proinflammatory mediators such as IL-6 and C-reactive protein (Figler et al., 2011). Deletion of the A_2B_AR gene and selective blockade of A_2B_AR in mice reduced hepatic glucose production and enhanced glucose disposal into skeletal muscle and brown adipose tissue (Figler et al., 2011). By contrast, other studies suggested an anti-diabetic role of A_2B_AR. Johnston-Cox and colleagues showed that A_2B_AR plays an essential role in high fat diet (HFD)-induced insulin resistance in mice, and mice lacking A_2B_AR displayed diminished glucose clearance and elevated insulin resistance and inflammatory cytokine production (Johnston-Cox et al., 2012). The underlying cellular mechanism here is mediated by A_2B_AR expressed in macrophages: reinstatement of macrophage A_2B_AR expression in A_2B_AR-null mice restored HFD-induced insulin tolerance and tissue insulin signaling to the level in control mice. The molecular mechanism involves A_2B_AR altering cAMP signaling and the levels of macrophage cytokine expression and secretion, and this regulates the levels of insulin receptor-2 and downstream insulin signaling (Johnston-Cox et al., 2014). Similar results were obtained by Csoka et al. (2014), who suggested that A_2B_AR plays a crucial role in sustaining glucose homeostasis and preventing insulin resistance under normal dietary conditions by regulating alternative macrophage activation. Insulin- and glucose-induced glucose clearance was impaired in A_2B_AR-knockout mice that were fed chow diet, and these knockout mice also exhibited a low level of physical activity, which might contribute to decreased insulin sensitivity in skeletal muscles. Csoka et al. also highlighted the complex role of A_2B_AR in regulating liver metabolism (Csoka et al., 2014).

## Conclusion

In this review, we have discussed certain general characteristics of A_2B_AR and have described multiple binding partners of the receptor, including α-actinin-1 and p105, whose interactions with the receptor were discovered recently. This identification of A_2B_AR-binding proteins will undoubtedly help enhance our understanding of the molecular and cellular functions of A_2B_AR; however, to date, fewer binding partners have been reported for A_2B_AR than for other AR subtypes. Several reasons might account for this: (1) Little attention was previously devoted to A_2B_AR because the receptor was long assumed, inaccurately, to be of lesser physiological relevance as compared with other ARs; (2) studies on A_2B_AR were hampered by a lack of useful biological tools such as specific agonists; and (3) novel experimental approaches such as mass spectrometry were not used to identify A_2B_AR binding partners.

Recent studies have considerably advanced our understanding of the critical role of A_2B_AR in the pathogenesis of human diseases, and this raises the possibility that A_2B_AR could be used as a potential target in the treatment of cancer, diabetes, or other diseases. However, opposing functions of A_2B_AR have been identified in several diseases. For example, A_2B_AR activation produces pro- and anti-tumoral effects and the receptor performs pro- and anti-inflammatory functions. These paradoxical effects are least partly contributed by the incompletely explored, agonist-independent activities of A_2B_AR, including its interactions with p105 (Sun et al., 2012), netrin-1 (Corset et al., 2000), ADA (Herrera et al., 2001; Pacheco et al., 2005), or other effector proteins in specific contexts. Moreover, the discrepant effects might be ascribed to different systems and conditions used for studying them, including cell types, animal models, time window of modulation of A_2B_AR activity, and the potential side effects of given agonists or antagonists. From a clinical perspective, these opposite effects of A_2B_AR make it highly challenging to decide whether agonists or antagonists should be used in pharmacological interventions for a given disease. Therefore, to effectively use A_2B_AR as a therapeutic target, studies must be conducted to elucidate precisely how A_2B_AR agonist-dependent and -independent functions modulate a particular pathological condition in a specific cellular setting and time window.

## Author contributions

All authors listed, have made substantial, direct and intellectual contribution to the work, and approved it for publication.

### Conflict of interest statement

The authors declare that the research was conducted in the absence of any commercial or financial relationships that could be construed as a potential conflict of interest.
